# Phylogenetic Analysis of Transmission Dynamics of Dengue in Large and Small Population Centers, Northern Ecuador

**DOI:** 10.3201/eid2905.221226

**Published:** 2023-05

**Authors:** Sully Márquez, Gwenyth Lee, Bernardo Gutiérrez, Shannon Bennett, Josefina Coloma, Joseph N.S. Eisenberg, Gabriel Trueba

**Affiliations:** Universidad San Francisco de Quito, Quito, Ecuador (S. Márquez, B. Gutiérrez, G. Trueba);; University of Michigan, Ann Arbor, Michigan, USA (G. Lee, J.N.S. Eisenberg);; University of Oxford, Oxford, UK (B. Gutiérrez);; Institute for Biodiversity Science and Sustainability, California Academy of Sciences, San Francisco, California, USA (S. Bennett);; University of California, Berkeley, Berkeley, California, USA (J. Coloma)

**Keywords:** Dengue, phylogenetics, whole-genome sequencing, urban, remoteness, transmission, vector-borne infections, Ecuador, viruses, dengue virus

## Abstract

Although dengue is typically considered an urban disease, rural communities are also at high risk. To clarify dynamics of dengue virus (DENV) transmission in settings with characteristics generally considered rural (e.g., lower population density, remoteness), we conducted a phylogenetic analysis in 6 communities in northwestern Ecuador. DENV RNA was detected by PCR in 121/488 serum samples collected from febrile case-patients during 2019–2021. Phylogenetic analysis of 27 samples from Ecuador and other countries in South America confirmed that DENV-1 circulated during May 2019–March 2020 and DENV-2 circulated during December 2020–July 2021. Combining locality and isolation dates, we found strong evidence that DENV entered Ecuador through the northern province of Esmeraldas. Phylogenetic patterns suggest that, within this province, communities with larger populations and commercial centers were more often the source of DENV but that smaller, remote communities also play a role in regional transmission dynamics.

Dengue virus (DENV) is a vectorborne tropical disease transmitted by *Aedes aegypti* and *Ae. albopictus* mosquitoes. Globally, an estimated 390 million cases occur per year, and 3.9 billion persons are at risk for infection (*1*). Dengue is endemic to 100 countries; southeast Asia is the most severely affected region, followed by the western Pacific and the Americas (*1*).

Dengue is considered an urban disease because transmission is often reported in areas with high population density. However, human mobility and active commerce have increased in classically defined rural sectors where population density is generally lower, influencing DENV transmission. DENV transmission in rural sectors is often reported to occur at similar rates as urban areas (*2*–*4*). More remote rural areas can also serve as a source for emerging febrile diseases, often through cases introduced through cross-border movement. For instance, in Laos, surveillance of fevers in rural areas provided evidence that more DENV serotypes were circulating in rural areas than in urban ones (*5*). Those introductions highlight the need for highly coordinated and timely arbovirus surveillance at the local and national level to enable early warning of potential DENV outbreaks among neighboring countries (*6*,*7*). In Asia, cross-border surveillance systems and networks such as UNITEDengue have been established to share data on disease outbreaks to monitor and control the disease more effectively (*8*).

DENV has been endemic to South America since the 1980s and to Ecuador since 1988. Generally, only 1 serotype circulates episodically within a region in South America, but all serotypes have circulated and reemerged in cycles (*9*,*10*). During 2010–2019, explosive epidemics were reported in the Americas; cases peaked at 3.1 million in 2019 (*11*) After a period of low prevalence in 2020, likely because of underreporting caused by the COVID-19 pandemic (*12*) and limited human movement between regions, Ecuador reported an increase of DENV cases in 2021.

Esmeraldas Province is located in the northern coast of Ecuador and shares a border with the Nariño department of Colombia. Fifty seven percent of the population lives in poverty and lacks basic needs such as potable water and garbage collection services (C. Robbins, unpub. data, https://digitalrepository.trincoll.edu/cgi/viewcontent.cgi?article=1857&context=theses). In a previous study conducted in rural communities in Esmeraldas during 2013–2014, we detected circulation of all DENV serotypes, whereas in the city of Esmeraldas, the largest city in the province, we detected only 2 serotypes (DENV-1 and DENV-2) (*13*). Those findings suggest that rural communities can act as a source of DENV transmission. High human mobility and levels of commerce reported in towns along the Colombia border suggest that DENV cases found in rural communities in Ecuador were likely introduced from Colombia (C. Robbins, unpub. data).

On the basis of this previous evidence, the aim of this study was to extract DENV nucleotide sequences obtained from serum samples from active DENV cases collected during 2019–2021 in Esmeraldas Province. We investigated the phylogenetic relationship of those sequences to DENV nucleotide sequences from throughout Ecuador to learn more about the role of rural DENV transmission dynamics in northwestern Ecuador.

## Materials and Methods

### Study Site

**Figure 1:**
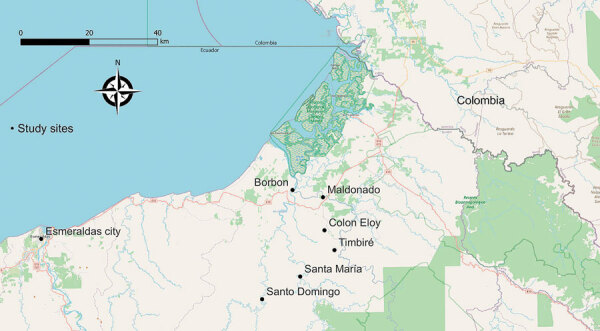
Locations of the 6 rural communities in Esmeraldas Province and the city of Esmeraldas for study of transmission dynamics of dengue in large and small population centers, northern Ecuador.

The study was conducted as part of an ongoing arboviral surveillance study in Cantón Eloy Alfaro, Esmeraldas Province, northwestern Ecuador. In brief, we selected 6 communities according to their gradient of remoteness: 2 remote riverine communities with no road access (Santa María and Santo Domingo), 3 communities with road access (Colón Eloy, Timbiré, and Maldonado), and 1 commercial center (Borbón) (Figure 1).

### Sample Collection

We collected data during May 2019–December 2021 by using active fever surveillance at the individual level. Except in the town of Borbón, community residents >2 years of age were invited to participate in prospective active surveillance. In Borbón, we invited a random sample of 500 households located in the town center to participate. In total, 1,460 households and 5,957 participants provided >1 month of active surveillance data from the study period of May 2019–December 2021. A group of community members trained to identify cases (brigadistas) conducted home visits weekly during the rainy season (June–October) and every 2 weeks during the dry season (November–December). The surveillance protocol began with brigadistas inquiring whether any household members had experienced fever, red eyes, or rash within the previous 7 days. When a symptomatic person was identified, the brigadista alerted the study nurse, who then followed up with a questionnaire asking about ≈21 symptoms associated with DENV and travel history. Participants experiencing diarrhea or upper respiratory symptoms were excluded. When a DENV-like illness was identified (fever plus rash, myalgia, arthralgia), a blood sample was immediately collected and a rapid diagnostic test performed (data not shown). Serum derived from the blood sample was stored in liquid nitrogen for transportation to the laboratory, where the samples were kept at −80°C until processing. The study was approved by the Bioethics committee at the Universidad San Francisco de Quito, the University of Michigan, and the Ecuadorian Ministry of Health.

### RNA Extraction, Reverse Transcription PCR, and Sequencing

We extracted viral RNA from 488 febrile serum samples by using the QIAamp Viral RNA Mini Kit (QIAGEN, https://www.giagen.com), according to manufacturer instructions. We used this RNA as a template for the triplex real time reverse transcription PCR (RT-PCR) Zika-Dengue-Chikungunya assay, which we performed to confirm infection and etiology (*14*). We serotyped 121 samples positive for dengue using a conventional RT-PCR with some modifications (*15*).

We considered 27 positive samples with a real-time PCR cycle threshold (Ct) value of <30 optimal for sequencing. We amplified a 20-µL aliquot of cDNA obtained using the Superscript IV protocol by multiplex PCR, combining 5 µL of 5X Q5 Reaction Buffer, 0.5 µL of 10 mM dNTPs, 0.25 µL of Q5 DNA polymerase, 15.25 µL nuclease-free water, and 1.5 µL primer pool A (10 µM). For DENV-1 and DENV-2, we added 1.5 µL primer pool B (10 µM) (*16*). We selected the samples that showed a 900 bp band in pool A and pool B mix for sequencing. Using 2.5 µl of PCR products from each pool diluted in 45 µl of PCR water, we prepared the cDNA MinION library by using a native barcoding kit (NB-114) with a ligation sequencing kit (LSK-109) following manufacturers’ instructions and loaded it into the MinION flow cell (FLO-MIN 106) (Oxford Nanopore Technologies, https://nanoporetech.com). We performed demultiplexing and adaptor removal with Porechop version 0.2.3_seqan 2.1.1 (https://github.com/rrwick/Porechop) and assembled the sequencing reads with a de novo assembly approach using Spades version 3.13.0 (http://cab.spbu.ru/files/release3.13.0/manual.html). Next, we mapped the reads in Minimap2 version 2.17-R941 (https://github.com/lh3/minimap2) against reference genomes for DENV-1 (GenBank accession no. NC_001477.1) and DENV-2 (accession no. NC_001474.2). The consensus sequence was generated with Samtools version 1.7 (http://www.htslib.org/doc/1.7/samtools.html) and the serotype was confirmed using Genome Detective Arbovirus Typing software Tool version 1.137 (*17*) and BLAST (https://blast.ncbi.nlm.nih.gov/Blast.cgi).

### Phylogenetic Analysis

In total, we generated 9 DENV-1 and 13 DENV-2 genome sequences from our field site and used them for the phylogenetic analysis (22 samples). In addition, we sequenced 5 samples from the national surveillance system at the reference hospital of the city of Esmeraldas, the capital of the province (3 DENV-1 and 2 DENV-2) (accession nos. SRR1593089–SRR15793115). The hospital samples were selected for patients residing in Esmeraldas on the basis of the patient’s clinical record. To augment the Ecuador data for phylogenetic analysis, we incorporated 22 sequences from GenBank, 21 representing El Oro Province (located in southern Ecuador) and 1 sample from Esmeraldas. To enable cross-country analysis, we included available GenBank sequences from Argentina, Brazil, Perú, Nicaragua, Belize, Venezuela, and Colombia. Before generating an alignment, we identified genomes from the National Center for Biotechnology Information by randomly selecting the results of a BLAST of our whole-genome sequences for each serotype separately. We generated alignments by using MAFFT on XSEDE version 7.471 (https://mafft.cbrc.jp/alignment/server), then visually inspected and refined them in Aliview version 1.28 (https://github.com/AliView/AliView).

We constructed a maximum-likelihood phylogenetic tree for each serotype by using RaxML-HPC Blackbox version 8.2.12 (https://cme.h-its.org/exelixis/web/software/raxml) under a general time-reversible substitution model with 1,000 bootstrap replicates and plotting the resulting tree in FigTree version 1.4.4 (http://tree.bio.ed.ac.uk/software/figtree). We explored the temporal signal of the datasets by performing a root-to-tip genetic distance analysis using TempEst version 1.5.3 (*18*) and rooted the trees using the R^2^ method. To explore the temporal patterns of DENV spread, we constructed Bayesian time-calibrated trees by using BEAST version 1.10.4 under a Hasegawa-Kishino-Yano substitution model and an uncorrelated lognormal relaxed molecular clock (*19*). We used a Bayesian skyline tree prior (*20*) with 10 groups and a piece-wise constant reconstruction. We ran Markov chain Monte Carlo chains for 100 million steps and logged trees every 1,000 steps; we discarded the first 10% of the trees as burn-in by using TreeAnnotator version 1.10.4 (https://beast.community/treeannotator). We assessed adequate mixing and convergence of model parameters, defined by effective sample sizes of >200, in Tracer version 1.7.1 (*21*) and summarized maximum clade credibility (MCC) trees for each run using TreeAnnotator version 1.10.4, summarizing node ages as median heights.

## Results

### Samples Collected and DENV Serotypes Identified

**Figure 2:**
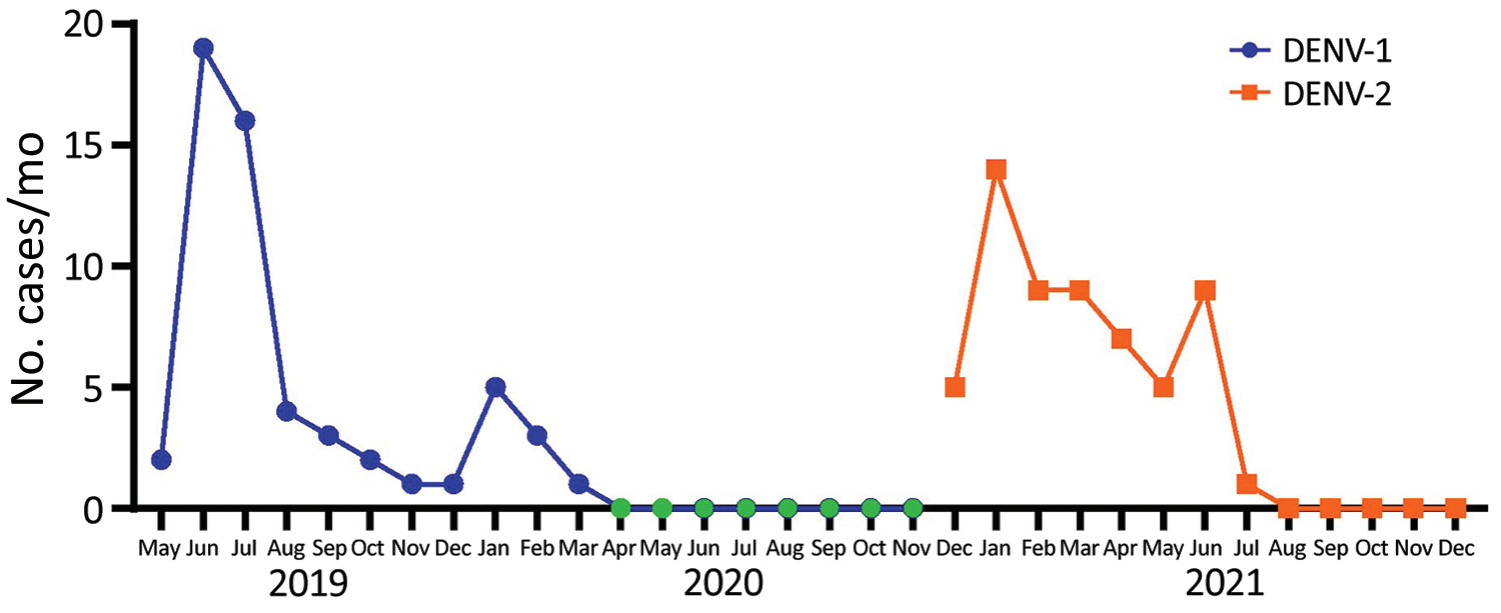
Number of febrile cases per month for DENV-1 (blue) and DENV-2 (orange) in study of transmission dynamics of dengue in large and small population centers, northern Ecuador, May 2019–December 2021. During April 2020–November 2020 (green), sampling was not conducted because of COVID-19 lockdown in Ecuador. DENV, dengue virus.

During May 2019–December 2021, we collected 488 serum samples from febrile patients during the household-based active fever surveillance study: 187 samples from remote riverine communities with no road access (146 from Santa María patients and 41 from Santo Domingo); 181 samples from communities with road access (61 from Colon Eloy, 78 from Maldonado, and 42 from Timbiré), and 120 samples from the commercial center, Borbón. Of the 488 samples, 121 were PCR-positive for DENV. A percentage of the samples were positive for chikungunya virus, as reported elsewhere (*22*). Common symptoms among patients were fever, headache, muscle aches, joint aches, chills, backache, and stomachache. Cases confirmed in June 2019 (19 cases) and July 2019 (16 cases) were all identified as DENV-1, whereas cases detected in January and March 2021 (12 cases) were all identified as DENV-2 (Figure 2). We did not obtain any samples during April 2020–November 2020 because of COVID-19 pandemic lockdown restrictions in Ecuador.

### Phylogenetic Analysis of DENV-1 Serotype in Ecuador

**Figure 3:**
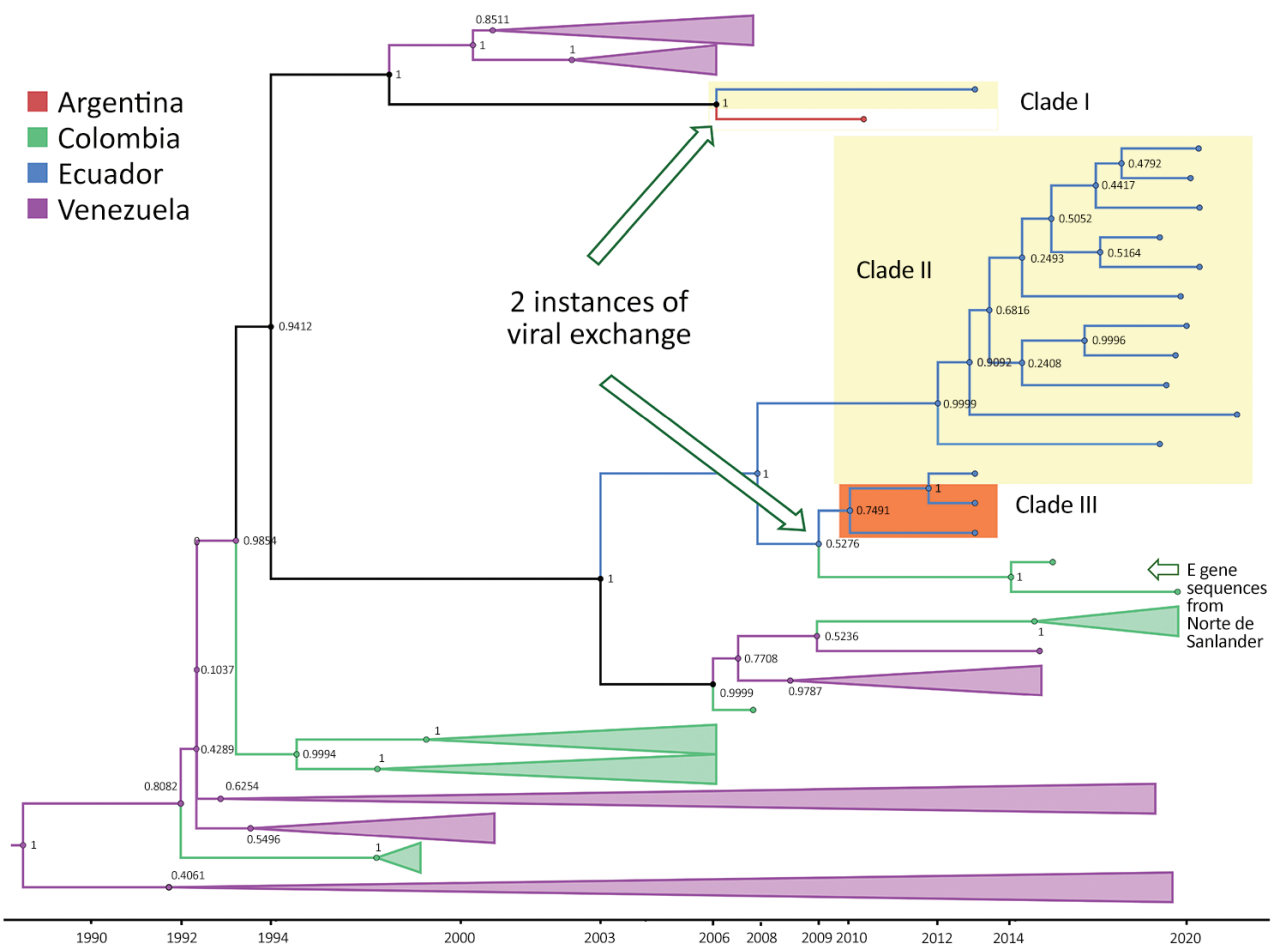
Maximum-clade credibility tree for dengue virus serotype 1 in study of transmission dynamics of dengue in large and small population centers, northern Ecuador. Tree was constructed using whole-genome sequences from Ecuador (blue), Colombia (green), Venezuela (purple), and Argentina (red) generated in BEAST software (https://beast.community). Within Ecuador, Esmeraldas Province samples are within the yellow rectangles and El Oro Province samples are within the orange rectangle, combined with E gene sequences from Norte de Santander department, Colombia. Posterior probabilities are shown in internal nodes. E, envelope.

**Figure 4:**
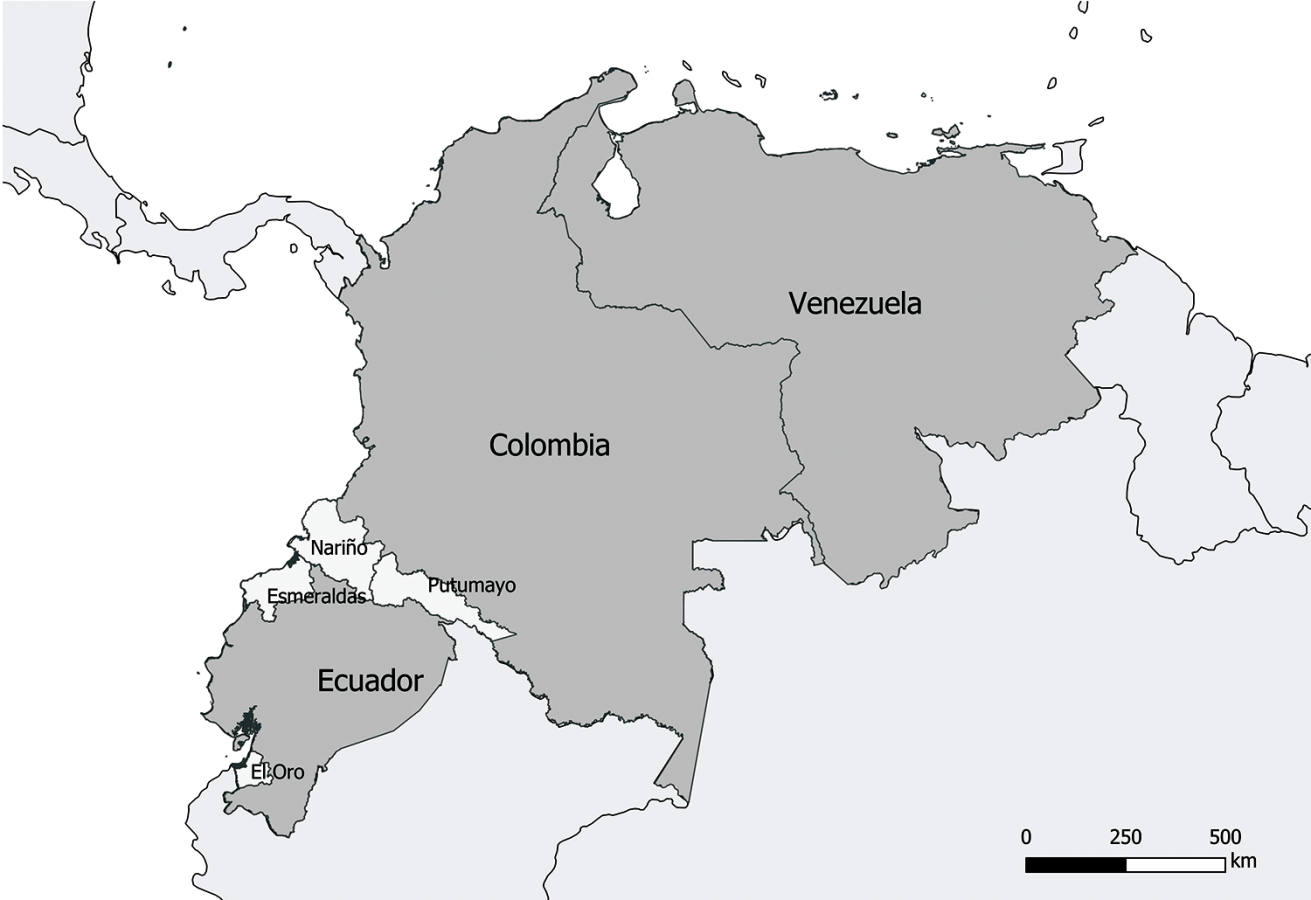
Location of El Oro Provinces in Ecuador, Nariño and Putumayo departments in Colombia, and Venezuela in relationship to Esmeraldas Province in study of transmission dynamics of dengue in large and small population centers, northern Ecuador.

The MCC tree for DENV-1 generated in BEAST software (http://beast.community) using whole-genome sequencing indicated at least 2 instances of viral variant exchange (viral exchanges) between countries. Those viral exchanges, inferred from the genetic similarities highlighted in the phylogeny, suggest that the strains were introduced into Ecuador. In one instance, a single Ecuador sequence collected in 2014 from Esmeraldas province (Figure 3, lower portion of clade I) shares a common ancestor with a sequence obtained in 2010 in Argentina. The theoretical most recent common ancestor of those 2 sequences was estimated to date back to 2006 and shared a common ancestor with viral sequences from Venezuela comprising the bulk of clade I (Figure 3). No additional sequences from this clade were detected in Ecuador, suggesting limited transmission after this potential introduction. The other DENV-1 viral exchange occurred no later than 2008 and was associated with the bulk of subsequent DENV-1 sequences from Ecuador. Those sequences formed 2 clusters, 1 from Esmeraldas Province and 1 primarily from El Oro Province that also included a sequence from Esmeraldas province (Figure 3, top portion of clade II and clade III). El Oro is located 600 km south of Esmeraldas Province (Figure 4). The introduction of those viruses into the rural communities of Esmeraldas Province is estimated to have occurred no later than 2012. Persistence of clade II lineage in Ecuador over time without the presence of other sequences from other countries suggests that Ecuador has sustained transmission that is continuing to evolve independent of external viral introductions.

### Phylogenetic Analysis of DENV-2 Serotype in Ecuador

**Figure 5:**
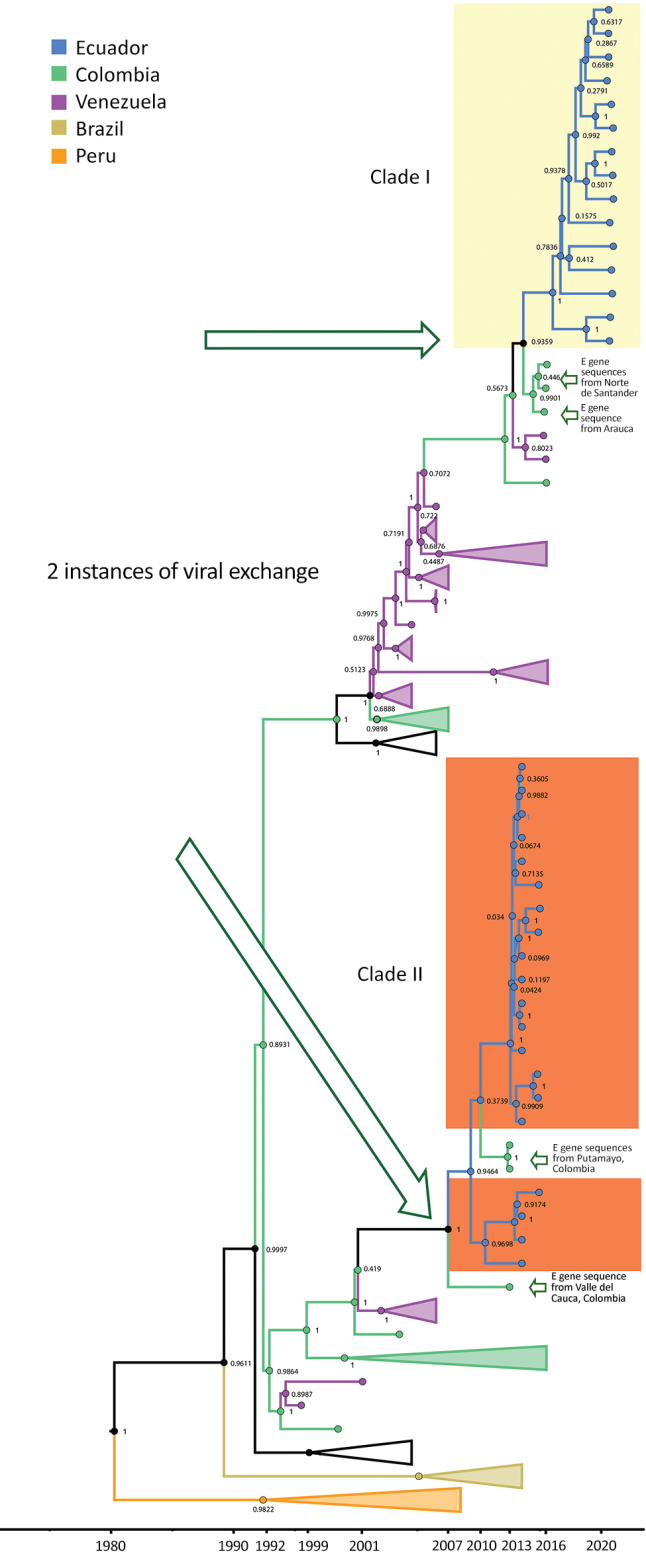
Maximum-clade credibility tree for dengue virus serotype 2 in study of transmission dynamics of dengue in large and small population centers, northern Ecuador. Tree was constructed using whole-genome sequences from Ecuador (blue), Colombia (green), and Venezuela (purple), combined with envelope gene sequences from Colombia departments (Nariño, Valle del Cauca, Putumayo, and Norte de Santander), and generated in BEAST software (https://beast.community). Within Ecuador, samples from Esmeraldas Province are within the yellow shaded area, and El Oro Province samples are within the orange shaded area. Posterior probabilities are shown in internal nodes.

The MCC tree for DENV-2 whole-genome sequencing (along with some partial genomes) also suggests >2 exchanges of distinct viruses among Ecuador, Colombia, and Venezuela. In one instance, a cluster composed of 20 sequences collected in 2014–2015 from Machala, a city in El Oro Province, shares a most recent common ancestor dating back to 2007 with sequences from Colombia (Figure 5, clade II). The closest related sequence to this Ecuador clade is a Colombia envelope gene sequence from Valle del Cauca collected in 2013. In addition, 2 envelope gene sequences from Colombia (Putumayo) sampled in 2013 clustered within the Ecuador clade II. The other instance of viral exchange is suggested by 2 clusters, a cluster of 15 Ecuador sequences collected from 2020–2021 active surveillance of rural communities in Esmeraldas Province, which share a most recent common ancestor dating back to 2013, and a cluster of sequences from Colombia (Figure 5, clade I). The sequences from Machala arrived in Ecuador earlier than those from Esmeraldas Province, possibly because the Machala study predates the Esmeraldas study (Figure 5).

### Phylogenetic Analysis of DENV-1 Serotype from Esmeraldas Province

**Figure 6:**
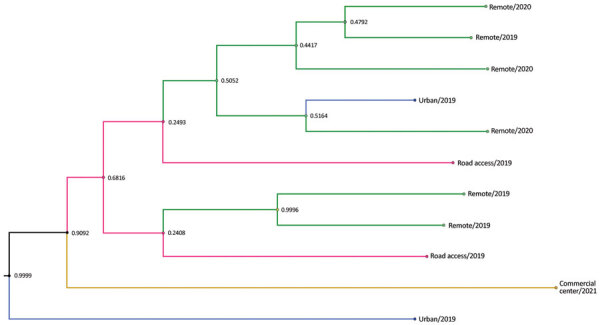
Subclade tree of dengue virus serotype 1 from rural communities of Esmeraldas Province in study of transmission dynamics of dengue in large and small population centers, northern Ecuador. Gradient of remoteness is classified as remote communities with no road access (green), communities with road access (pink), commercial center (yellow), and urban (blue). Subclade nodes are labeled with posterior probabilities generated in BEAST software (https://beast.community).

Focusing on the DENV-1 serotype from our study in Esmeraldas Province (Figure 3, clade II), we labeled sequences on the basis of their gradient of remoteness: remote communities with no road access (Santa María and Santo Domingo), communities with road access (Colon Eloy, Timbiré, and Maldonado), the commercial center (Borbón), and the large population center of Esmeraldas Province (Esmeraldas city) (Figure 6). This DENV-1 subclade tree does not show distinct clustering by gradient of remoteness, although it does support the hypothesis that the cities of Esmeraldas and Borbón are sources of the viruses circulating in the smaller communities in our study site. A sequence from the city of Esmeraldas collected in 2019 (root position posterior node probability 0.9999) and a sequence from the Borbón hospital collected in 2021 (root position posterior node probability 0.9092) shared a most recent common ancestor dating back to 2012 (Figure 6). The descendants of those 2 ancestral sequences are found in remote communities and communities with road access; however, an Esmeraldas city sequence collected in 2019 derived within this cluster is suggestive of a subsequent exchange back into the large population center.

### Phylogenetic Analysis of DENV-2 Serotype from Esmeraldas Province

**Figure 7:**
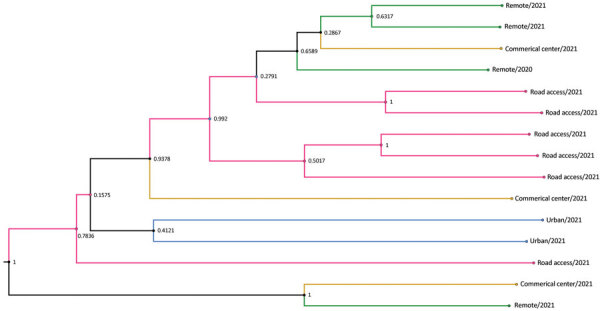
Subclade tree of dengue virus serotype 2 from rural communities of Esmeraldas Province in study of transmission dynamics of dengue in large and small population centers, northern Ecuador. Gradient of remoteness is classified as remote communities with no road access (green), communities with road access (pink), commercial center (yellow), and urban (blue). Subclade nodes are labeled with posterior probabilities generated in BEAST software (https://beast.community).

The DENV-2 Ecuador lineage from Esmeraldas Province demonstrates clustering by community (Figure 7, clade I). We also found strong support (0.9378) for the commercial center (Hospital of Borbón) and for those communities with road access (Colon Eloy and Timbire) being intermediate sources (0.992) of DENV-2 strains moving between the city of Esmeraldas and 2 remote communities (Santa Maria and Santo Domingo). The most recent common ancestor dates back to 2018 for a subset of the remote samples. Although much of the data support viral movement from cities and commercial centers to more remote communities, our phylogeny also supports movement from a remote community (Santo Domingo) to the commercial center of the region (Borbón) (Figure 7).

## Discussion

Human mobility is one of the main determinants of DENV transmission (*23*) because the flight range of *Aedes* mosquitoes is minimal (*24*). The social dynamics of the region combined with our results suggest that Esmeraldas Province is an entry point for DENV strains arriving in Ecuador from Colombia. Legal and illegal commerce (mostly by sea) between Esmeraldas Province and Colombia is robust (*25*,*26*) and occurs primarily in small, mostly rural communities with weak customs controls that favor informal and illegal commerce and irregular human migration.

Our data suggest that DENV-1 entered Ecuador no later than 2009 and moved to at least 2 Coastal provinces in Ecuador (Esmeraldas and El Oro). Sequences found in those provinces were most closely related to nucleotide sequences from Colombia, but they also showed similarity to sequences from Venezuela (Figure 3). Another DENV-1 sequence shares a theoretical most recent common ancestor from 2006 with an Argentina sequence and also shares high similarity with sequences from Venezuela (Figure 3). The first DENV-2 introduction might have occurred earlier than 2009, and the virus circulated in Ecuador until 2015. This DENV-2 strain was most closely related to Colombia strains circulating until 2013 (Figure 5). The second introduction probably occurred no later than 2013, resulting in an Ecuador lineage that persisted at least until 2021. Those DENV-2 sequences showed high similarity to both Colombia and Venezuela sequences.

The perspective that large tropical cities are the primary source of DENV (and other *Ae. aegypti* mosquito–transmitted viruses) has resulted in establishment of surveillance centers in large urban centers, but in Ecuador, social (and commercial) dynamics suggest that DENV is also entering through small rural community centers and subsequently disseminating to the cities. Although most of our data suggest that DENV is brought to remote communities from urban centers, we also found instances of DENV movement from remote communities to larger commercial centers (e.g., Esmeraldas and Borbon). Smaller communities with road access probably provide a bridge between more remote communities and larger commercial centers, such as Esmeraldas and Borbón, thereby serving as intermediate sources. Large population centers, such as Esmeraldas, are hubs for public transportation and therefore may become the epicenter of viral spread (*27*–*30*). Further studies are needed to understand how a heterogeneous landscape (including biological, social, and environmental factors) among communities in the region drive epidemic dynamics (*31*).

One reason that DENV transmission has remained endemic to regions such as Ecuador (in contrast to the diminished transmission of Zika and chikungunya viruses) is that there is continual serotype and lineage replacement to which cross immunity is only partial (*31*,*32*). For example, the Ministry of Health reported a 2.8-fold increase in the number of dengue cases from 2014 to 2015 (15,031 to 42,459 cases) (*32*,*33*). This increase was likely caused by introduction of a new lineage of DENV-2 from Colombia to Ecuador, leading to the replacement of the previously dominant lineage that had been circulating since 2012 (Figure 5). The replacement of lineages has been reported in several studies associated with outbreaks linked to the introduction of novel lineages (*34*,*35*) and has long been considered a part of DENV transmission dynamics in endemic and hyperendemic regions (*25*,*26*,*36*). Those replacements might be fueled by viruses displaying novel antigenic variations for which the local population lacks immunity (*37*) or by viral variants with higher fitness (*38*). Another reason for the observed increases might be that cases reported by the Ministry of Health are other *Ae. aegypti* mosquito–transmitted arboviruses, such as chikungunya virus (*22*).

Several surveillance gaps should be considered when interpreting our data, including minimal historical specimens, lower resolution in rural sites than in urban sites, and limited country-specific data from countries in South America (including Ecuador). Although those gaps are reflective of working in a resource-limited setting, our data also reflect a unique contribution to dengue transmission dynamics in a resource-limited setting. Additional gaps in our data are a result of the COVID-19 pandemic, which might have resulted in our missing DENV cases and potential DENV introductions. Ecuador’s countrywide state of emergency (March 16–April 24, 2020) and subsequent restrictions forced us to stop or sharply curtail our active household-based surveillance study (Figure 2, period of no surveillance shown in green). This state of emergency similarly affected the government’s passive surveillance program.

We believe our conclusions are robust for several reasons. First, we are using complete genome sequences. Second, we obtained posterior probabilities by using a Bayesian Skyline analysis that provides us with a level of confidence for our phylogenetic relationships. Third, our results are biologically plausible and are supported by the social dynamics we observed.

For example, evidence is ample that Colombian viruses are ancestral to and a potential source of Ecuador DENV (Figures 3, 5). Colombia has numerous, very active Caribbean ports that likely serve as entry points to South America for the virus (*39*,*40*; B. Gutierrez, University of Oxford, pers. comm., 2023 Feb 14). In contrast to previous reports that suggest migration from Venezuela drives DENV lineage introduction into Ecuador (*39*), we believe its role is minimal. Ecuador does not share a border with Venezuela, and 75% of immigrants from Venezuela travel through Colombia (a 5-day journey). In addition, the main border crossing point in the highlands (Rumichaca, Ecuador) (*41*) is a region with no *Ae. aegypti* mosquito activity. A recent report, however, shows that migrants from Venezuela play a vital role in the introduction of DENV to Colombia (*42*), so further introductions to Ecuador through the Colombia border are likely.

Although our findings are consistent with what is known about the social dynamics of the study region, samples collected through community-based surveillance studies and previously reported strains were opportunistically gathered, usually from clinical settings. DENV cases are likely underestimated in Ecuador. Viral genomic characterization from countries such as Colombia and Venezuela are similarly limited, further adding uncertainty to our capacity to estimate the precise source and direction of exchanges.

Cases in smaller population centers are increasingly recognized as playing a vital role in broader DENV transmission dynamics (*43*). Our phylogenetic analyses in Ecuador shed light on those dynamics. Esmeraldas Province might be a key site from which DENV is introduced, amplified through local transmission, and then spread throughout Ecuador by human travel and movement of infected mosquitoes. Our results underline the need for coordinated efforts in DENV strain monitoring and control across national borders. Ministries of Health should intensify dengue surveillance in neglected remote regions, especially along national borders. Surveillance can also elucidate how DENV diffuses and becomes endemic, which requires an understanding of how large population centers affect viral dynamics regionally, and whether smaller, more remote communities represent a silent reservoir of ongoing dengue circulation.
